# A Case of Laying Hens Mycosis Caused by *Fusarium proliferatum*

**DOI:** 10.1155/2023/5281260

**Published:** 2023-04-30

**Authors:** Ramziya M. Potekhina, Evgenya Yu. Tarasova, Lilia E. Matrosova, Nail I. Khammadov, Alexander M. Saifutdinov, Olga K. Ermolaeva, Svetlana A. Tanaseva, Nailya N. Mishina, Gali N. Nigmatulin, Aisylu Z. Mukharlyamova, Sergey Yu. Smolentsev, Eduard I. Semenov

**Affiliations:** ^1^Federal Center for Toxicological, Radiation and Biological Safety, Kazan 420075, Nauchnyi Gorodok-2, Russia; ^2^Mari State University, Lenin Square 1, Yoshkar-Ola City 424000, Russia

## Abstract

In this article, we present the first case report of a chicken mycosis caused by *F. proliferatum* occurred on a private farm in the Russian Federation. Lesions on the skin of the legs and scallops were reported. The object of this study was samples of feed and pathological material from sick hens-layers. Mycological analysis included determination of the total number of fungi (TNF) and identification and determination of the toxicity and pathogenicity of the isolates. The identification of the isolate was carried out taking into account direct microscopy, morphological features, and the method of molecular genetic analysis. Microscopic fungi of the genus *Penicillium* and *Rhizopus* were isolated by mycological analysis of the feed. The test feed was nontoxic. Mycological examination of pathological material (scrapings from the combs and affected legs) identified an isolate of *Fusarium proliferatum*, which showed toxicity on biological objects (protozoa, rabbits) and pathogenicity (white mice). Dermal application of *F. proliferatum* suspension was accompanied by reddening of the rabbit skin. Intraperitoneal injection of fungal spores caused mycosis in white mice. Polymerase chain reaction (PCR) made it possible to identify this type of microscopic fungus (*F. proliferatum*) with high accuracy in the samples under study. The research results allow us to consider *F. proliferatum* as a cause of poultry disease against the background of predisposing factors in the form of desquamation of the stratum corneum of the skin against the background of immunosuppression and metabolic disorders caused by an imbalance in the diet.

## 1. Introduction

*Fusarium* is a group of the most common plant pathogens found throughout the world that are detrimental to crop production. *F. proliferatum* is a very important member of the *Fusarium* genus and is capable of infecting many economically important crops including wheat, peas, corn, rice, asparagus, date palm, garlic, onion, pineapple, and banana [1–3]. *F. proliferatum* spreads mainly with seeds and crop residues and biosynthesizes a large number of mycotoxins (bovericin, moniliformin, fusaric acid, and highly toxic fumonisins) [4–6].

This globally widespread fungal pathogen can survive in many ecological niches, but the optimal environment is a warm and humid climate, as well as loamy soils [7]. Such characteristics indicate the high plasticity and excellent adaptability of *F. proliferatum* to changing environmental conditions [8, 9]. *Fusarium* infections are very difficult to fight as effective fungicides are not available, so multistep actions are required, including plant and soil protection and reduction of inoculation [10, 11]. In addition, the accumulation of secondary metabolites of *Fusarium* in plant tissues and their possible harmful effects on human and animal health is an additional problem in effective plant protection [12, 13]. The best strategy for controlling an infection is to produce plants that are resistant to the pathogen [14, 15]. Recently, reports have begun to appear in the literature that unusual fungal species, such as *F. proliferatum* and others, may be underestimated as agents, causing mycotic infections, including mycoses in animals and humans. Molecular phylogeny in a study [16] showed that 23 *Fusarium* species, distributed over eight species complexes, were associated with veterinary mycoses. The most common infections in animals were eye infections, followed by dermatomycosis and onychomycosis. In addition, the authors conclude that *F. redolens* caused cutaneous granuloma in a cat in California, which is only the second report of this soil fungus associated with mycotic infection.

Two cases of noninvasive fungal rhinosinusitis are described [17], which developed due to the *F. proliferatum* disease. They were the first to associate this fungal pathogen with maxillary fungus balls of odontogenic origin [18]. With an emphasis on genetic identification of *F. proliferatum*, the histopathological features of granulomatous feline pododermatitis with fusarium infection were first described. There has also been a reported case of infection of *F. proliferatum* in humans via a punctured finger from a plant [19]. In the article [20], *F. proliferatum*, identified by EF-1ɑ DNA sequencing, is described as an important new species causing fungal keratitis in China, where it was isolated out of 877 cases with a frequency of 17.7%. However, the authors note that the role of *F. proliferatum* in this disease is not well understood. A case of nonhaual hyalogyphomycosis caused by *Fusarium proliferatum* has been reported in an immunocompetent patient. Irregular hyalophomycosis due to *Fusarium* spp. is rare, with an incidence of about 3% in Switzerland [21]. In Japan, out of 11 cases of hyalophomycosis caused by *Fusarium* spp., there were three infections with *F. proliferatum*, accompanied by the development of subungal onychomycosis [22].

Our paper indicates that *F. proliferatum* is capable of causing mycosis in chickens. However, summarizing the literature data, we can conclude that mycoses of animals and humans caused by *F. proliferatum* have not actually been studied.

## 2. Materials and Methods

### 2.1. Research Objects

The studies were carried out in the laboratory of mycotoxins of the Federal State Budgetary Scientific Institution “Federal Center for Toxicological, Radiation, and Biological Safety” (Russian Federation). The objects of the study were samples of feed (compound feed PK 1-2-63) and pathological material from sick and dead birds from a private poultry farm. Laying hens of the LOHMANN cross at the age of 38–64 weeks were kept in cages of 12 individuals each. The composition of the diet is presented in Table 1.

### 2.2. Determination of the Content of Mycotoxins in Feed

Spectral analysis conducted by ultra-high performance liquid chromatography using an Agillent 1290 Infinity II chromatograph, a Zorbax SB-C18 chromatographic column (150 × 2.1 mm, 5 *μ*m), and an AB SCIEX Triple Quad 5500 mass spectrometric detector. Two methods were used to detect the following mycotoxins: T-2 toxin, HT-2 toxin, T-2 triol, diacetoxycisprenol, neosolaniol, deoxynivalenol, nivalenol, fuzarenone-X, moniliformin, bovericin, fumonisins B1, B2, B3, aflatoxin B1, G1, sterigmatocystin, ochratoxin A, patulin, zearalenone, alternariol, and tentoxin [23, 24].

### 2.3. Determination of Microscopic Fungi

Isolation of microscopic fungi from the feed was carried out by the method of successive dilutions with further inoculation on an agar Czapek medium (glucose 30 g, sodium nitrate 2.0 g, monosubstituted potassium phosphate 1 g, magnesium sulfate 0.5 g, potassium chloride 0.5 g, ferrous sulfate 0.01 g, distilled water 1 l, and agar-agar 20 g). Isolation of microscopic fungi from pathological material was carried out by inoculation on Sabouraud agar medium (fermentative dry peptone-7.0 g, soy flour hydrolyzate-3.0 g, hydrated crystalline glucose-40.0 g, clarified autolyzed yeast extract-4.0 g, and microbiological agar for dense medium-12.0 g).

For mycological analysis, food was taken after uniform mixing 10.0 g of the prepared food was placed in a flask with 100 ml of sterile 0.1% solution of the surfactant Tween-80 in distilled water. The resulting suspension with the ratio of feed and solvent 1 : 10 was shaken for 15–20 min on a shaker. Then, they were diluted with sterile distilled water (the ratio of feed and water is 1 : 10, 1 : 100, 1 : 1000, and 1 : 10000), and 1 ml of each of these dilutions was added to the surface of the nutrient medium in Petri dishes. The inoculations were incubated in a thermostat at a temperature of 22–25°C for 5–7 days. The number of spores in 1 g of feed and the species composition of fungi were determined. The total number of colonies grown in the dishes of the main dilution was taken as 100% and the percentage of individual species of fungi was calculated accordingly.

The calculation of the total amount of fungi in 1 g of the product was carried out according to the following formula:

(1) $$\begin{array}{c} X=\frac{\sum c}{0,1*V_{1}+0,01*V_{2}+0,001*V_{3}}\text{,} \end{array}$$

where *X* is the total number of fungi, expressed by the number of colony-forming units (CFU) in 1 g of the product; ∑*c*-the sum of colonies on all plates, calculated in the inoculations of all three consecutively diluted suspensions; *V*_1_-volume of suspension 1 (dilution 10^−1^); *V*_2_-volume of suspension 2 (dilution 10^−2^); *V*_3_-suspension volume 3 (dilution 10^−3^).

Scrapings were made from the affected foci of legs and scallops in birds using a sterile scalpel (at the sites of infection on the border of healthy and affected parts of the keratinized skin). The previously affected area was treated with 96% ethyl alcohol. Keratinized elements from legs and scallops were applied to agar media of Sabouraud and Czapek. Mycological studies of feed included identification, determination of the toxicity of isolated isolates on the test culture of *Paramecium caudatum* and on the skin of a rabbit, and determination of pathogenicity was carried out on white mice.

### 2.4. Identification of Fungi

The genus of the isolate was determined taking into account the morphological characters using a light microscope (Olimpus CX23), which were compared with those in the fungus identifier, as well as with our long-term photo collection of fungi. The preparations were prepared from a 5-day old culture of the isolate grown on agar medium [25]. A drop of fixing liquid (Amman lactophenol: distilled water 1 part, lactic acid 1 part, glycerin 2 parts, and phenol 1 part) was applied to a glass-alcohol-free glass slide using a microsyringe, then a piece of mycelium was taken with a mycological hook and layered on it, covered with a cover glass, then looked under a microscope at ×10 and ×40 magnifications.

At the next stage, the species was confirmed by PCR. When creating a tool for the indication of microscopic fungi of the *Fusarium* genus by PCR, the following resources were used: National Center for Biotechnology Information resources (https://www.ncbi.nlm.nih.gov); “BLAST,” basic local alignment search tool utility (https://blast.ncbi.nlm.nih.gov/Blast.cgi); Vector NTI 9.1.0 program, bioinformatics analysis methodology is similar to our earlier papers [26, 27]. The material for the research was the washings from the nutrient media of colonies of microscopic fungi. For the study, 500 *μ*L of wash was taken, which was centrifuged at 5,000 rpm in a mini spin Eppendorf centrifuge; for further isolation of nucleic acids, 200 *μ*L of sediment was taken. Isolation of nucleic acids (NC) was carried out by the method of magnetic sorption with a set of reagents “MAGNO-sorb” option 100–200 (“AmpliSens”) according to the manufacturer's instructions. For each PCR amplification, the following composition of the reaction mixture was used, per sample: 1.5 *μ*l of 25 mM MgCl_2_ solution; 1.5 *μ*l of 2.5 mM dNTP solution; 1.5 *μ*l 10x PCR buffer; 0.5 *μ*l of 10 pM PCR probe solution; 10 pM solution of forward and reverse primers, 0.5 *μ*l; 0.5 *μ*l Taq polymerase; 5 *μ*l DNA and 3.5 deionized water. For PCR, we used reagents manufactured. The final volume of the reaction mixture was 15 *μ*L.

For PCR amplification, 2 different loci were used, characterized by the presence of marker sequences in the studied microscopic fungi. The first locus is represented by the nucleotide sequence of the marker locus *Fusarium proliferatum* strain PP74 18S ribosomal RNA gene, internal transcribed spacer 1, 5.8S ribosomal RNA gene, internal transcribed spacer 2, and 28S ribosomal RNA gene, region (https://www.ncbi.nlm.nih.gov/nuccore/FJ884099.1/). Oligonucleotide primers for amplification of this locus are additionally designated “ITS.” The second locus is represented by the nucleotide sequence of the marker gene *Fusarium proliferatum* strain 9,233 translation elongation factor 1-alpha gene (https://www.ncbi.nlm.nih.gov/nuccore/JX869031.1).

Oligonucleotide primers for amplification of this locus have the additional designation “*α*.” RT-PCR was performed using the following primers: forward primer F pro ITS-AGCGGAGGGATCATTACCGAGTTTAC, reverse primer F pro ITS–AAAGTTTTGATTTATTTATGGTTTTACTCAGAAGTTACAT, and probe F pro ITS–Cy5-ATTGTTGCCTCGGCGGATCAGCC-BHQ3; forward primer F pro *α*–TGCGGTGGTATCGACAAGCGA, reverse primer F pro *α*-GAGCGGGGTAGCAGGCACGT, and probe F pro *α*–Rox-CTCTGCCCACCGATTTCACTTGCGATT-BHQ2. All oligonucleotide primers for determining marker loci of *Fusarium proliferatum* have the designation (except for indicating the type of oligonucleotide primer) “F pro.” RT-PCR (real-time PCR) was carried out on a C1000 amplifier with a CFX96 optical unit. The amplification program was as follows: (i) DNA denaturation at 95°C for 2 minutes; (ii) 5 cycles consisting of 10 seconds at 95°C and 30 seconds at 60.5°C; (iii) 40 cycles consisting of 10 seconds at 95°C and 30 seconds at 60.5°C; detection of the PCR result (fluorescence) occurs in each of 40 cycles of the third stage of PCR, at 60.5°C through the Rox channels and Cy5.

### 2.5. Biotesting on Protozoa *Paramecium caudatum*

The express method for determining the toxicity of fungal isolates on protozoa is based on the extraction of toxic substances with the subsequent effect of these extracts on the Paramecium. The mycelium of micromycetes (5 days old) was poured with distilled water in a ratio of 1 : 1 by volume, and then mixed and incubated at a temperature of 10°C for 24 hours. Two drops of the extract from fungal cultures were applied to a glass slide using a Pasteur pipette, and one drop of the medium with protozoa was added. The behavior of the Paramecia was observed under a Carl Zeiss Stemi 2,000 C stereoscopic microscope. If the protozoa did not die on the slide within 3–5 minutes, they were placed in a Petri dish on a circle with filter paper moistened with water to prevent the droplets from drying out.

The criterion for determining the sensitivity of paramecium to toxic substances is the time from the onset of exposure to the test extract to the death of protozoa, which is ascertained on the basis of cessation of movement, deformation, or decay. If there is no death of the Paramecia, then they are monitored for no more than 2 hours. In the case when at least a part of the Paramecia during this time stops moving, the observation continues until the moment of death of all specimens. Many toxic metabolites cause the death of protozoa in the period from 1 to 20–30 minutes, low-toxic ones-up to 1-2 hours. The survival rate of Paramecia was determined by the following formula:

(2) $$\begin{array}{c} N=N2÷N1\times 100\text{,} \end{array}$$

where *N* is the arithmetic mean (of five tests) value of the amount of paramecium at the end of the experiment after 1 hour of exposure, pcs; *N*1 is the arithmetic mean (of five tests) value of the number of parameters at the beginning of the experiment, pcs; 100 is the translation of the result into percent.

An isolate is considered toxic if 0 to 39% of protozoa die; slightly toxic from 4069%, nontoxic from 70100%.

### 2.6. Biotesting on Rabbits

When setting a skin test on rabbits, the mycelial film of the fungus grown on a nutrient medium was rubbed to a mushy state and applied with a glass rod to a clipped 6 × 6 cm area in the thigh area (the hair was cut off until completely exposed), rubbed lightly. For control, one bare skin area with a similar size 6 × 6 was used, to which the extract was not applied. In order to prevent licking of the extract applied to the skin, a collar was put on the rabbit's neck, which was removed 3 days after the first application. On the next day after applying the extract, the reaction according to the state of the skin was taken into account: (+) First degree*-*redness of the skin, increased sensitivity-redness, soreness, and peeling; (++) Second degree*-*redness, soreness, peeling, thickening of the skin, and small rash in the form of bubbles of darkish coloration; (+++) Third degree-redness, pronounced thickening of the skin, soreness and folding, superficial necrosis, and scab formation; (++++) Fourth degree-severe swelling in the form of a roller on the lower border of the site, dry necrosis, and/or a continuous thick scab. Rabbits were not euthanized.

### 2.7. Biotesting on White Mice

When setting up a bioassay on white mice, in order to determine pathogenicity, 2 groups of animals were formed (*n* = 10). The control group was injected with physiological saline intraperitoneally in a volume of 0.5 ml. The experimental animals were injected with 0.5 ml of a micellar suspension of a 5-day culture of the isolate with a concentration of 0.5 and 1 billion spores. The animals were observed for 14 days. The dead animals were dissected immediately; control and surviving mice were dissected at the end of the experiment using a humane method. The surviving mice were euthanized by dislocation of the cervical vertebrae after light anesthesia with ether for anesthesia (inhalation). At autopsy, inoculations were made-prints from internal organs on Czapek agar to isolate micromycetes.

### 2.8. Statistical Analysis

Statistical processing was performed using the STATISTICA 10 software (StatSoft, USA). Data are presented as the mean values ± standard deviation.

### 2.9. Bioethics

Experiments on animals were carried out according to strict rules regarding care and experimental use (rules of the Ministry of Agriculture of the Russian Federation and international rules), and were approved by the local ethics committee of the Federal State Budgetary Scientific Institution “FCTRB-VNIVI” (Kazan, Russia, no. 2/01.22.2019).

## 3. Results and Discussion

**Figure 1:**
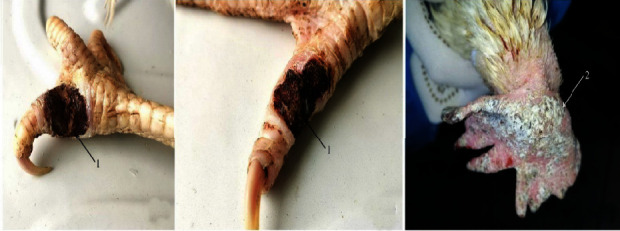
Lesions of the legs and combs (1-amina with the formation of scab-like crusts, 2-focal necrotic lesions of the comb).

In the spring of 2019, on one of the private farms of the Russian Federation, the death of laying hens at the age of 6 months, ulcerative-necrotic lesions of the combs were reported on the toes with the formation of scab-like crusts, painful on palpation, and signs of pododermatitis. On clinical examination, lameness was noted. The birds were losing weight. The described clinical signs were progressive in nature;-they began with small pinpoint rashes and then intensified. The disease has been registered in poultry farms for more than 2 years, but less than 3 years.  Figure 1 shows the affected combs and legs of birds.

We would call a problem considerable as the poultry farm contains more than 3 million layers at the same time. Clinical signs had been noted at the age of 120 days, though pathoanatomical changes in a liver and in kidneys could be visualized from 70–90 days age. Thus, it is possible to state pathology at 2.78 million layers, which is the prevalence approximately 92.7% of the livestock.

In addition to vivid signs of damage to the legs and scallops, changes were also noted in the internal organs. At autopsy, toxic liver degeneration was reported, the liver was flabby, yellow (“fatty liver”), the condition of the mucous membranes of the glandular and muscular stomachs was satisfactory, but the intestinal mucosa, especially the small intestine, was hyperemic, swollen, and easily detached; the pancreas was without visible changes. A decrease in the size of the spleen was a characteristic of the ureters-uric acid diathesis. Infectious (bacterial and viral) and invasive diseases (ectoparasites-ticks) were previously excluded in the interregional veterinary laboratory.

We conducted mycotoxicological and mycological studies of feed and pathological material. Taking into account the results of preliminary studies and clinical signs of the disease, we have chosen the mycological direction of searching for the cause of the disease.

**Figure 2:**
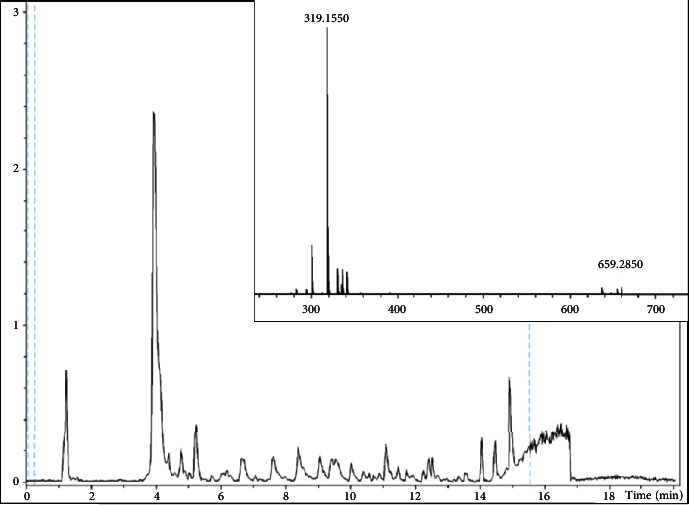
Chromatogram of a compound feed sample; the inset shows the mass spectrum of zearalenone.

Mycotoxins T-2 toxin, moniliformin, bovericin, fumonisin B1, fumonisin B2, fumonisin B3, zearalenone, alternariol, and tentoxin were detected; however, the detected concentrations were below the acceptable levels and insignificant (Table 2 and Figure 2).

**Figure 3:**
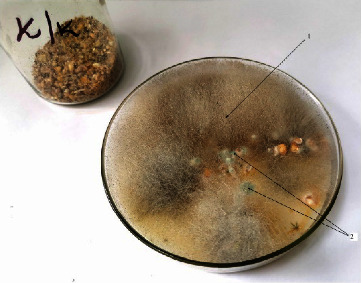
Growth of isolates (1-*Rhizopus* sp.; 2-genus *Penicillium* sp.).

During biotesting on paramecia and rabbit skin test, the studied food was nontoxic. Mycological analysis of the food revealed fungi of the genus *Penicillium* sp., *Rhizopus* sp. (Figure 3).

The total number of fungi was 3.1 × 10^3^ CFU/g of feed. This does not exceed the standard established in Russia (5.0 × 10^4^ CFU/g of feed) and indicates the absence of mold growth. The detected isolates of fungi of the genus *Penicillium* and *Rhizopus* did not have toxicity for *P. caudatum*; the survival rate of the test culture was 83.7 ± 2.5%. The test on rabbit skin also did not reveal that their toxic properties-skin redness, soreness, or peeling were not reported.

**Figure 4:**
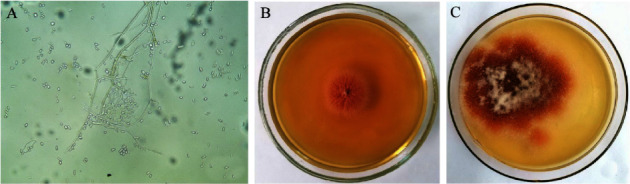
*Fusarium proliferatum* ((a)-Microscopy of the preparation at x40; (b) 3-day culture of the fungus; (c) 7-day-old culture of the fungus).

During mycological analysis of pathological material, *F. proliferatum* was isolated from the affected tissues of birds. It should be noted that other fungi were not found, when inoculated on nutrient media. The mycelium of the culture was pink; the colonies of the fungus are clearly delineated, with a uniform growth. Colonies are fast-growing; mycelium is branched on conidiophores. Microconidia are numerous, mostly pear-shaped, elongated, and truncated. Macroconidia are fusiform-sickle-shaped with a more pronounced pedicle at the base in the aerial mycelium, arranged in chains, the links of which are collected in false heads, oval in shape with 0-1 septum. Chlamydospores are intermediate, forming singly, in pairs in clusters and in chains. The sclerotia had a plexus of hyphae of a horn-like consistency (Figure 4).

**Figure 5:**
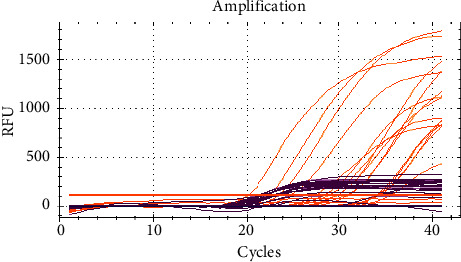
Amplification of marker loci for the indication of microscopic fungi of the *Fusarium* genus: amplification of marker loci of *Fusarium proliferatum*.

Genetic confirmation of the species *F. proliferatum* was performed by amplification of the “internal transcribed spacer” “translation elongation factor 1-alpha” genes (Figure 5).

Each genetic marker was indicated by an individual fluorescent label, so the Cy5 detection channel was used for the “internal transcribed spacer” gene (shown in the graph as purple lines); for the translation elongation factor 1-alpha gene, the Rox detection channel was used (the graph shows in the form of orange lines). The isolate of fungi of the *Fusarium* genus, identified by PCR as *Fusarium proliferatum*, had toxicity, the death of protozoa was more than 91 ± 3.8%. The reaction on the rabbit's skin was positive-redness, soreness, and peeling were observed. When administered intraperitoneally to white mice at a dose of 0.5 and 1 billion spores, it showed pathogenicity with the death of 75 and 90% of the animals within 7 days. Oppression of animals was noted, the coat was disheveled, the mice showed thirst, accompanied by abundant drinking, and refusal to feed. An autopsy was performed after the death of the animals. On visual inspection of the corpses, the coat is disheveled. The mucous membranes of animals are pale pink with no visible changes. The animal carcasses are exhausted; the heart and lungs are without visible changes. On examination of the stomach, small foci of hemorrhage are visible. When the stomach is cut, a small amount of fodder and mucus is observed. When examining the colon, foci of hemorrhage are also observed. Basically, the intestines are empty and contain no fodder. The kidneys are bean-shaped; in the section, the border of the cortical and medullary layers is smoothed. Blood was inoculated on agarized Czapek media and by the method of imprinting a part of the parenchymal organs, spleen, and liver, followed by incubation at a temperature of 25°C for 5 days. An isolate of *F. proliferatum* was isolated in the organs. Probably, multiple passages of the mycosis pathogen from bird to bird that occurred naturally over a long period of time increased the contagiousness of the isolate.

The principles of management of this farm are to exclude any components of animal origin (Table 1). Deficiency of animal proteins leads to metabolic disorders, disruption of normal liver function, including the function of synthesis of significant immunoglobulins, which creates the prerequisites for the development of immunosuppression, a decrease in the detoxification function of toxic and potentially toxic xenobiotics that enter the food. Violation of metabolic processes contributes to the violation of skin keratin, the stratum corneum becomes loose. This fungus was not found in feed; probably the causative agent of mycosis was introduced earlier. The study of the pathogenicity of the isolate allows us to consider it in our case not as a saprophytic fungus, but as a pathogen that is the cause of the disease in poultry against the background of predisposing factors in the form of metabolic disorders and immunosuppression. The long and progressive nature of the disease also suggests a fungal origin of the disease. In confirmation of this, there is the fact that the owners have been practicing the exclusion of animal components from the diet for more than 7 years, but such a clinical picture has not been previously observed in birds.

Some mycotoxins, such as trichothecenes, can cause both external damage to the skin and cause negative consequences in internal organs [28] which were observed during autopsy. The state of immunosuppression of the body was confirmed by a decrease in the size of the spleen. In mycoses, characteristic destructive processes are often seen on the skin [29–31], which we observed on the limbs and scallops. Mycotoxins can alter the metabolism of the pathogen, which can change the outcome of an infectious disease [32, 33]. Although there is information about the mutual enhancement of mycotoxins upon combined intake of animals [34, 35], nevertheless, given the detected concentrations, in this case they cannot be considered as the cause of chicken disease.

By analogy with paper, we carried out a study using direct microscopy, further cultured and identified the isolate morphologically and by PCR as for *F. proliferatum*. Direct microscopy revealed chlamydioconidia and hyphae in the same way as in the paper [36]. The isolate had typical morphological properties. The identification of *Fusarium proliferatum* is considered successful with positive amylation of both specific markers. A similar approach for the indication of microscopic fungi (from two loci) is proposed [37, 38]. The clinical signs observed in this case, including emaciation and weight loss, suggest a decrease in immune function as a predisposing factor for infection, and the disturbance of the stratum corneum, in combination with crowded poultry and moisturizing of the wounds, enable the development of *F. proliferatum* [39].

According to *Fusarium* spp., an increasingly important cause of infections in people, especially those with weakened immune systems. A type of hyalogyphomycosis, fusarium can occur in both healthy and immunocompromised people. In healthy birds, microscopic fungi usually invade soft tissue by traumatic inoculation [40]. These infections take the form of onychomycosis, keratitis, endophthalmitis [41], and mycetoma [42]. In patients with neutropenia caused by cytotoxic chemotherapy, *Fusarium* spp. can cause systemic *Fusarium*. Skin infections associated with *Fusarium* spp. are rare in healthy people. As a rule, skin infections of *Fusarium* develop in immunocompromised patients, usually during a systemic infection with a virulent strain of *Fusarium* [43]. Moreover, the infection can progress both locally, causing extensive tissue destruction, and spread hematogenously. Our literary search showed only a few cases of onychomycosis in healthy people [44, 45]. In these cases, *F. proliferatum* infection may have occured through contact with colonized plants or soil. We think that infections caused by *Fusarium* spp. are becoming an increasingly important cause of infections, especially not only in immunocompromised people but also in animals and poultry.

Violation of the norms for the stocking density of chickens in the workshops and the violation of air exchange, due to insufficient ventilation, led to injuries and the ingestion of spores of mold fungi of the *Fusarium* genus on the affected skin in the area of the legs. The favorable temperature and humidity conditions for the field isolates contributed to the rapid spread of the disease. Since almost all types of fungi are opportunistic pathogens, an important condition for the onset of the disease was a decrease in the body's resistance caused by feeding an unbalanced diet and violation of the conditions for keeping poultry.

## 4. Conclusion

Thus, given the danger of microscopic fungi of the *Fusarium* genus, capable of infecting organs and tissues, causing pathological processes with weakened immunity, it is necessary to observe zootechnical requirements, strictly take into account the norms of keeping birds, take preventive measures in a timely manner, control humidity and air exchange in workshops, and timely carry out mycological analysis of feed, as well as monitor the quality of storage of grain in bunkers in order to avoid mold growth. *F. proliferatum* infections present a difficult therapeutic challenge, even for an experienced mycologist. We also emphasize the importance of accurate species diagnosis for decision-making on further treatment strategies for such cases. There is a lot of information on fungal infections acquired as a result of trauma [44, 45]; this report is, to our knowledge, the new case report associated with the occurrence of mycosis caused by *F. proliferatum* in chickens.

## Figures and Tables

**Table 1:** Laying hens feeding diet.

Components	Content in the diet (%)
Peas	3.00
Sunflower cake SP 34%, SK 20%	18.10
Feed flour	2.00
Sunflower oil	0.72
Lysine monochlorohydrate 98%	0.11
DL-methionine 98.5%	0.09
L-threonine 98%	0.07
Table salt	0.12
Monocalcium phosphate	0.30
Limestone flour (grains 1–3 mm)	5.85
Shell 2–5 mm	3.00
Bicarbonate of soda	0.05
Allzyme Vegpro	0.05
Premix 1% “Vitomek”	1.00

**Table 2:** Content of mycotoxins in compound feed.

Determined indicators	Test results (*μ*g/kg)
T-2 toxin	4.18 ± 0.12
HT-2 toxin	Not found
T-2 triol	Not found
Diacetoxycisprenol	Not found
Neosolaniol	Not found
Deoxynivalenol	Not found
Nivalenol	Not found
Fuzarenone-X	Not found
Moniliformin	<5.80
Bovericin	3.64 ± 0.13
Fumonisin B1	10.84 ± 0.06
Fumonisin B2	<5.80
Fumonisin B3	<5.80
Aflatoxin B1	Not found
Aflatoxin G1	Not found
Sterigmatocystin	Not found
Patulin	Not found
Zearalenone	1.84 ± 0.16
Ochratoxin A	Not found
Alternariol	32.11 ± 0.07
Tentoxin	15.89 ± 0.06

## Data Availability

The data used to support the findings of this study are included within the article.
